# Motion Patterns Under Multiple Constraints and Master–Slave Control of a Serial Modular Biomimetic Robot with 3-DOF Hydraulic Muscle-Driven Continuum Segments

**DOI:** 10.3390/biomimetics10050278

**Published:** 2025-04-29

**Authors:** Yunrui Jia, Zengmeng Zhang, Junhao Guo, Yong Yang, Yongjun Gong

**Affiliations:** 1 Naval Architecture and Ocean Engineering College, Dalian Maritime University, Dalian 116026, China; jyr0104@dlmu.edu.cn (Y.J.); junhaog@dlmu.edu.cn (J.G.); wateryy@dlum.edu.cn (Y.Y.); gyj@dlmu.edu.cn (Y.G.); 2 Key Laboratory of Rescue and Salvage Engineering Liaoning Province, Dalian 116026, China

**Keywords:** biomimetic robot, water hydraulic artificial muscle, motion pattern, master–slave control

## Abstract

Soft modular biomimetic robots, driven by flexible actuators, are extensively used in various fields due to their excellent flexibility, environmental adaptability, and isomorphism. However, existing flexible modules typically possess no more than two degrees of freedom for structural limitations. In this study, a three-degree-of-freedom biomimetic segment driven by water hydraulic artificial muscles (WHAMs) and supported by springs was proposed, achieving integrated and modular design. The continuum robot composed of this segment can execute earthworm-, snake-, and elephant trunk-biomimetic motion modes based on operational environmental constraints. During long-distance operational tasks, distinct segments of the continuum robot can adopt varying biomimetic configurations to meet specific requirements. The closed-loop control characteristic tests were conducted on a single segment to evaluate its motion characteristics. The isomorphic master controller was designed based on the motion range of a single segment, with the maximum bending angle deviation between the master controller and biomimetic segment not exceeding 4°, and the system demonstrating favorable stability.

## 1. Introduction

With the rapid advancement of robotics technology, biomimetic robots have garnered significant attention [1,2,3]. Compared with conventional rigid robots, biomimetic robots demonstrate remarkable potential across domains such as medical rehabilitation [4,5], disaster rescue [6], and industrial automation [7,8]. Their advantages stem from inherently flexible locomotion, enhanced environmental adaptability, and improved safety profiles, which collectively enable novel applications.

A modular robot consists of multiple identical modules, where the design process focuses on the structure and functionality of individual modules. This approach ensures the modules’ structural integrity and rapid interconnectivity [9]. Many researchers have utilized this advantage to design modular robots for different tasks. Inspired by earthworm locomotion, Tang et al. [10] developed a novel modular robot designed for narrow environments. The system utilizes shape memory alloy (SMA) springs, whose controlled activation/deactivation enables individual module extension and deflection, while a synergistic mechanism integrating rigid links and gear trains ensures kinematic efficiency by mitigating postural redundancy. Mu et al. [11] developed modular articulated snake-like robots featuring dual-degree-of-freedom joints. Each joint’s outer frame incorporates a spherical structure, which is coupled to the actuation system via a two-stage gear reduction mechanism. Experimental results demonstrate that the proposed robotic systems can effectively execute typical gait patterns and obstacle avoidance maneuvers across varied terrain conditions.

Inspired by the biological architecture and locomotor principles of earthworms, Fang HB et al. [12] developed a pipeline robot. The system integrates servo–motor-actuated tendons and spring-loaded flexible bands within each modular segment, biomimetically replicating the antagonistic action of longitudinal and circular muscles in earthworms. This design enables alternating segmental contraction and elongation, generating retrograde peristaltic waves that facilitate effective horizontal locomotion and vertical climbing within confined tubular environments. Experimental results demonstrate that the eight-segment prototype achieved these capabilities across various pipeline. Wang N et al. [13] developed a hexagonal prism-shaped soft biomimetic actuator leveraging silicone rubber’s nonlinear hyper-elasticity, with design principles inspired by the musculoskeletal architecture and undulatory locomotion of terrestrial annelids. Different inflation methods enable it to achieve different motion modes within the pipeline. By leveraging reconfigurable modular designs in conjunction with adaptive control strategies, modular robots achieve dynamic environmental adaptation and mission versatility, thereby significantly enhancing their adaptability to unstructured environments and scalability across diverse tasks.

Modular robotic systems exhibit distinct locomotor strategies under varying spatial constraints in engineering applications. Specifically, robots designed for operations in confined spaces typically utilize radial expansion mechanisms for stable environmental anchoring. Their locomotion is achieved through the coordinated, sequential activation of multiple modular units [14]. When radial deformation alone cannot generate sufficient anchoring forces in the presence of environmental boundary confinements, modular robots typically employ coordinated multi-segment deformation to establish contact interfaces with surrounding structures. This bio-inspired strategy enables both anchoring stability and load-bearing capacity enhancement through optimized inter-segmental normal force distribution [15,16]. When operating in unknown or unstructured environments, modular robotic systems commonly utilize elephant trunk-inspired biomimetic architectures to achieve dexterous manipulation and adaptive obstacle avoidance. This design paradigm, informed by the kinematic principles of an elephant trunk, enables hyper-redundant motion in unstructured spaces through compliant segment interaction and distributed sensing [17]. During real-world operations, the operational scenarios and locomotion requirements of continuum robots dynamically evolve as the system interacts with its environment. To address this challenge, we present a novel continuum robot featuring 3-DOF modular segments, which enables multi-modal kinematic configurations for adaptive operation across various environments.

In engineering practice, master–slave control architectures are commonly employed to enhance human–machine collaboration efficiency and mitigate operational complexity. The research on master–slave control methods mainly includes isomorphic master–slave control and non-isomorphic master–slave control. Yang et al. [18] adopted an incremental master–slave control algorithm utilizing a position–orientation separation method, which overcomes the problems of isomorphism and workspace inconsistency between the master and slave while ensuring the same motion trend between them. Meanwhile, a safety assurance mechanism was integrated into the master–slave control architecture to eliminate the situation where the slave robot arm moves violently in a short period of time resulting from operator mis-operation. Ai Y et al. [19] investigated the master–slave control architecture for heterogeneous surgical robots in minimally invasive surgery. Through systematic analysis of the master–slave configuration in MIS robotic systems, they proposed an incremental control methodology based on decoupling of gesture and positional control, effectively resolving the workspace incompatibility between master and slave manipulators arising from structural heterogeneity. Wang LQ et al. [20] developed a novel 6-DOF isomorphic master–slave submarine manipulator for deep-water operational environments. A mapping framework was systematically established for the master–slave system. Comprehensive experimental evaluations demonstrated that the end-effector trajectories of the master and slave manipulators exhibited congruence. Zheng et al. [21] proposed a novel master robot that utilizes a modular design approach. Based on the isomorphic systems of the master and slave robots, this design enables simple, intuitive, and stable control of the slave robot. An isomorphic master control allows for simple, intuitive, and stable control of robots, whereas non-isomorphic master control units facilitate the incorporation of safety mechanisms and motion range limitations, thereby ensuring the stable operation of the master–slave control system. Therefore, by combining the advantages of isomorphic and non-isomorphic master–slave control strategies and based on the structure and motion range of the three-degree-of-freedom segment, this paper designs an isomorphic master control unit and imposes motion constraints to ensure the stability of the master–slave control system.

This article proposes a continuum robot segment designed to be compact and modular, facilitating the formation of a continuum robot. The robot adapts to various environmental constraints based on the segment’s motion, degrees of freedom, and functions, and can pass through a confined space and operate in a structured environment.

The rest of this article is organized as follows. Firstly, the structural composition, integration, and modular design of the three-degree-of-freedom segment are presented. Subsequently, the motion forms of the continuum robot composed of this segment are analyzed, achieving earthworm-, snake-, and elephant trunk-biomimetic behaviors under varied constraints. The motion characteristics under these constraints are further detailed. Finally, isomorphic master–slave control system was designed for single-segment motion, and the system’s effectiveness and stability were experimentally demonstrated.

## 2. Three-Degree-of-Freedom Segment

**Figure 1:**
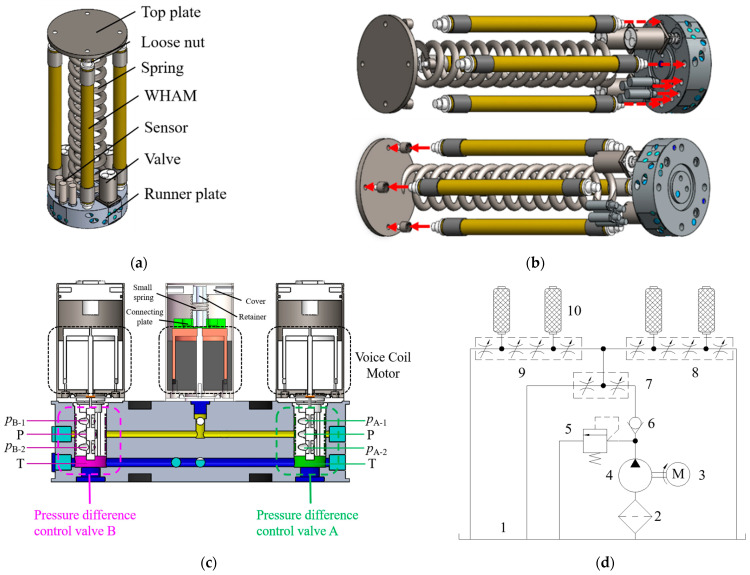
The structure and hydraulic circuit of a single segment. (**a**) Integrated prototype of biomimetic segments; (**b**) biomimetic segment assembly; (**c**) internal sectional view of the runner plate; (**d**) hydraulic circuit 1 water tank, 2 filter, 3 electric motor, 4 water hydraulic pump, 5 relief valve, 6 check valve, 7 total pressure control valve, 8 pressure difference control valve A, 9 pressure difference control valve B, 10 WHAM.

In order to achieve modular and integrated design of robot segments, an integrated solution is proposed, which tightly integrates the WHAMs inlet, valve, and sensor into the runner plate, as shown in Figure 1. Figure 1a,b show in detail the component composition, assembly, and series connection between segments. Specifically, a single segment is driven by four WHAMs and supported by spring. Four WHAMs form two pairs of orthogonal WHAM groups. One end of the WHAM is connected to the runner plate, while the other end is connected to the top plate or the runner plate of the next segment through a loose nut. Four pressure transmitters are integrated on the runner plate to monitor the working pressure of WHAMs. This design not only facilitates the assembly and disassembly of the segment, but also achieves the flexibility and scalability of the modular design. The valve is tightly connected to the runner plate through the insertion, as shown in Figure 1c. The pressure difference control valves A and B in Figure 1c correspond to the pressure difference control valves A and B, respectively, in Figure 1d. The pump has a rated flow rate of 3.5 m^3^/h, and its operating pressure does not exceed 8 MPa. During the working process, the fluid medium first passes through the total pressure control valve and then divides into two paths to enter pressure difference control valve A and pressure difference control valve B. The structures of pressure difference control valve A and pressure difference control valve B are completely identical and consist of one pressure inlet, one return outlet, and two pressure outlets. The two pressure outlets of pressure difference control valve A or pressure difference control valve B are, respectively, connected to two WHAMs, and these two WHAMs are a group. When the valve spools of pressure difference control valve A and pressure difference control valve B are centrally positioned, the working pressures at both outlets equalize (with four WHAMs operating at their initial working pressure), and adjusting the valve’s spool position induces contraction of the segment. When the valve spools of valves A and B deviate from the central position, the outlet pressures diverge, creating a pressure differential across the paired WHAMs. This results in one WHAM contracting while the other extends, producing segmental bending. The cornerstone of modular design resides in the runner plate, where the hydraulic circuitry is fully integrated. This integration enhances both compactness and operational efficiency of the modular segment. Inter-module hydraulic connections are established through a spring-shaped pipeline, which maintain flow continuity while improving system reliability and serviceability. This configuration enables a flexible, high-efficiency hydraulic system that satisfies the modular integration requirements of continuum robots. Additionally, the design facilitates system scalability and future upgrades. Physical parameters pertaining to segment are detailed in Table 1.

## 3. Robot Configuration and Motion Patterns

**Figure 2:**
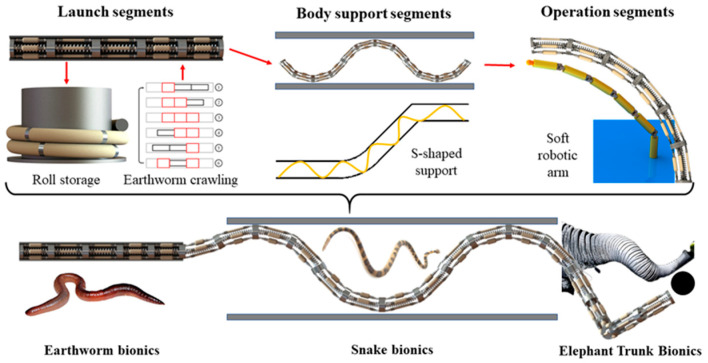
Engineering application.

The continuum robot enables diverse biomimetic motions inspired by animal kinematics, facilitating a broad spectrum of engineering applications. Correspondingly, robots exhibit different biomimetic movements under varying constraint conditions, as illustrated in Figure 2. When the segment is under radial constraint, the robot can achieve a worm–like peristaltic motion through the contraction and expansion of the segment. This configuration enables applications in narrow environments such as pipeline inspection/maintenance and power cable deployment through small-diameter conduits. When contraction and expansion of WHAMs fail to provide adequate anchoring force, the continuum robot can establish an S-shaped support configuration through coordinated bending of multiple segments. This serpentine posture facilitates snake-like locomotion patterns, enabling the robot to adapt its geometry to environmental constraints while maintaining operational stability. During the execution of search or object manipulation tasks in unstructured space, the entire continuum robot can emulate the kinematic behavior of an elephant trunk while operating as a compliant robotic manipulator. In long-distance robotic operations, environmental constraints vary across mission segments. Taking underwater shipwreck detection as an example, continuum robots utilize their flexibility to store. Under radial constraints from the launch tube, the robot enters the cabin. Due to the cabin’s large internal structural space, the robot employs snake-like crawling with S-shaped support. Upon entering the designated cabin, the robot executes biomimetic motion at its distal section, resembling an elephant’s trunk, while maintaining fixed S-shaped support segments to perform exploration, grasping, and other operational tasks. The robot then completes the underwater sunken ship detection mission.

## 4. Radial Constraint

**Figure 3:**
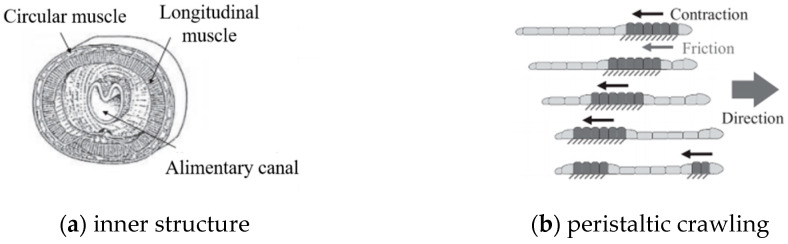
Peristaltic crawling pattern of an earthworm [22].

Earthworms, annelid organisms with segmented bodies, achieve crawling motion through coordinated muscle activity across their segments. Figure 3a illustrates the segment’s internal anatomy, showing circular muscles and longitudinal muscles. Their movement mechanism (Figure 3b) operates as follows. During contraction phases, circular muscles relax while longitudinal muscles contract, causing setae to extend and anchor the segment. During elongation phases, circular muscles contract and longitudinal muscles relax, with setae retracting to propel the body forward. This alternating pattern of axial contraction/radial expansion and axial elongation/radial contraction between segments generates forward locomotion.

Multi-segment robots achieve in-pipe crawling through contraction/extension of WHAMs, emulating earthworm biomimicry via synchronized segmental state transitions. Four WHAMs operate under unified working pressure, contracting by overcoming axial compression spring forces to generate body shortening. As working pressure increases, these WHAMs exhibit three distinct operational phases:

Pre-contact Preparation Phase: WHAMs overcome spring resistance while maintaining radial clearance from the pipe wall;

Threshold Contact Phase: WHAMs establish initial pipe wall contact yet exhibit negligible interaction force;

Active Anchorage Phase: WHAMs maintain firm pipe wall engagement, generating both anchoring traction and structural load capacity.

Assuming that four hydraulic artificial muscles maintain a cylindrical configuration during contraction and that the working part experiences no axial displacement, the force–balance equation between a single segment and a pipeline under different contact states can be expressed as follows:

(1) $$4F_{\mathrm{mlow}}-k_{s}\cdot (h_{0}+h_{\mathrm{low}})=\Delta F$$

(2) $$4F_{\mathrm{mhigh}}-k_{s}\cdot (h_{0}+h_{\mathrm{high}})=\Delta F$$

(3) $$4F_{\mathrm{mrhigh}}\cdot \mu =\Delta F$$

where, *F*_mlow_ represents the axial contraction force of the WHAM under low working pressure; *h*_0_ signifies the pre-compression of the spring; *h*_low_ represents the compression variation of the spring when the WHAM is subjected to low working pressure; *F*_mhigh_ denotes the axial contraction force of the WHAM under high pressure; *h*_high_ represents the compression variation of the spring when the WHAM is under high working pressure; Δ*F* signifies the axial load of the module; *F*_mrhigh_ represents the normal force between the WHAM and the pipeline inner wall under high working pressure; *μ* denotes the friction coefficient between the WHAM and the pipeline inner wall; and *k*_s_ is the spring stiffness, which is related to its material and size. When the robotic segment’s structural parameters and pipeline inner-wall surface properties remain constant, the interfacial friction generated between WHAMs and the pipeline wall is exclusively governed by two factors: the working pressure of the WHAMs, and the pipeline material’s intrinsic frictional characteristics. This frictional resistance exhibits a direct positive correlation with increasing WHAM operating pressure. The test results revealed that within a pipeline with an inner diameter of 180 mm, the segment generates up to 8000 N of axial load capacity at a WHAM working pressure of 1.5 MPa. Under pipeline constraints, robots utilize the contraction and expansion functions of their segments to perform biomimetic crawling movements akin to those of earthworms, thereby simplifying the motion functions of the segments. Studies have already been conducted on the load-bearing capacity [23], motion speed [24], pipe diameter adaptability, and elbow adaptability [25] of earthworm-mimicking robots under such constraints, and these aspects will not be re-analyzed here. The robot composed of these modules demonstrates exceptional load-bearing capacity under pipeline constraints, as evidenced by experimental testing.

## 5. Analysis of Contact Force of S-Shaped Support

### 5.1. Bending Moment of a Single Segment

When a multi-segment continuum robot assumes an S-shaped support configuration, the segments interacting with environmental constraints generate reaction forces to maintain equilibrium. Under the condition that torque transmission occurs between two constraint-engaged segments, the bending moment experienced by each individual segment and the entire robotic system remains equivalent. In the study conducted by Jia et al. [26], a single-segment mechanical model was analyzed.

(4) $$\begin{array}{l} M_{\mathrm{spring}}+M=\frac{1}{2}\pi D_{0}^{2}k_{F}\Delta p\{a[{\left(1-k_{\varepsilon }\cdot \varepsilon_{C}\right)}^{2}+k_{\varepsilon }^{2}\cdot \varepsilon_{\varphi }^{2}]-b\}\cdot r\cos\frac{\varphi }{2}\\ -\pi D_{0}^{2}k_{F}p_{0}ak_{\varepsilon }\varepsilon_{\varphi }(1-k_{\varepsilon }\varepsilon_{C})\cdot r\cos\frac{\varphi }{2} \end{array}$$

(5) $$\varphi =\frac{k_{s}nD_{\mathrm{spring}}(16M_{\mathrm{spring}}-k_{\tau }\pi \tau_{0}d_{\mathrm{spring}}^{3})}{16GI_{p}}$$

where *φ* is module deflection angle, *k*_F_ is the contraction force coefficient, *k*_ε_ is the contraction ratio coefficient, *k*_s_ and *k*_τ_ represent the rod length coefficient and the stress coefficient of spring, *τ*_0_ signifies the pre-stress of spring, *D*_spring_ signifies the pitch diameter of spring, *d*_spring_ denotes the wire diameter of the spring, *G* denotes the shear modulus of the spring material, *I_p_* represents the polar moment of inertia of the spring wire section, *M* is module bending moment load, and *ε_C_* and *ε_φ_* are contraction ratio caused by initial working pressure *p*_0_ and working pressure variation. *ε_φ_* is express as:

(6) $$\varepsilon_{\varphi }=\frac{2r\sin\frac{\varphi }{2}}{L_{0}}$$

Given the compact design presented in this study, we assume the WHAMs maintain perpendicular orientation relative to both the runner plate and the top plate. Additionally, for small angular displacements, the sine of the angle is approximated as equivalent to the angle itself. The bending moment in a single direction can be expressed as:

(7) $$\begin{array}{l} M=\frac{1}{2}\pi D_{0}^{2}k_{F}\Delta p\{a{\left(1-k_{\varepsilon }\cdot \varepsilon_{C}\right)}^{2}-b\}\cdot r-\pi D_{0}^{2}k_{F}p_{0}ak_{\varepsilon }(1-k_{\varepsilon }\varepsilon_{C})\cdot r^{2}\frac{\varphi }{L_{0}}\\ -\frac{1}{16}k_{\tau }\pi \tau_{0}d_{\mathrm{spring}}^{3}-\frac{GI_{p}\varphi }{k_{s}nD_{\mathrm{spring}}} \end{array}$$

**Figure 4:**
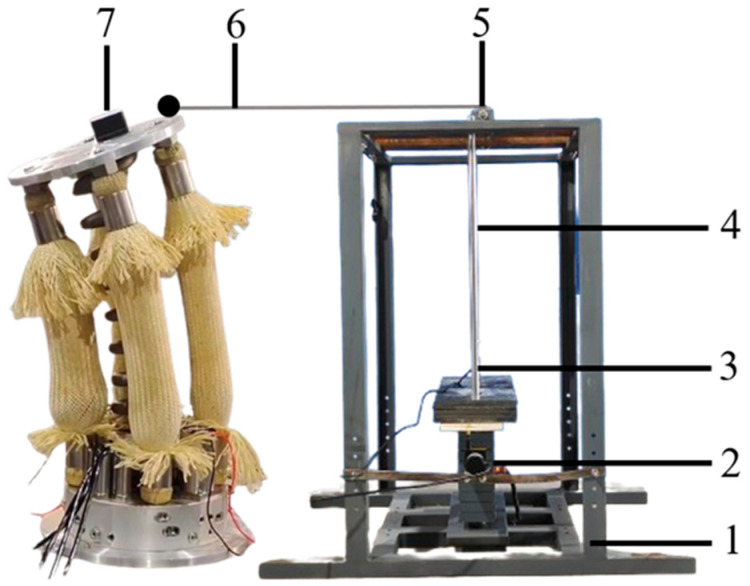
The load experiment system of segment. 1. Fixed frame, 2. load block, 3. force sensor, 4. guide rods, 5. fixed pulley, 6. steel wire, 7. angle sensor.

Under specific parametric configurations of the segmental structure, the bending moment capacity is governed by the initial working pressure, working pressure variation, and bending angle. The performance evaluation of springs and hydraulic artificial muscles was conducted, with the relevant parameters presented in Equation (7) and corresponding values provided in Table 2. To evaluate the load-bearing capacity of the segment, the experimental setup was designed and implemented, as illustrated in Figure 4. The load block was placed in a fixed frame and constrained by two guide rods. The top plate of the module was connected to an end of steel wire, and the other end of the steel wire was wrapped around a fixed pulley and connected to the load block through a force sensor. The bending moment generated by the load block on the fixed end of the segment was the bending moment load of the segment. The technical parameters of the sensor are shown in Table 3.

**Figure 5:**
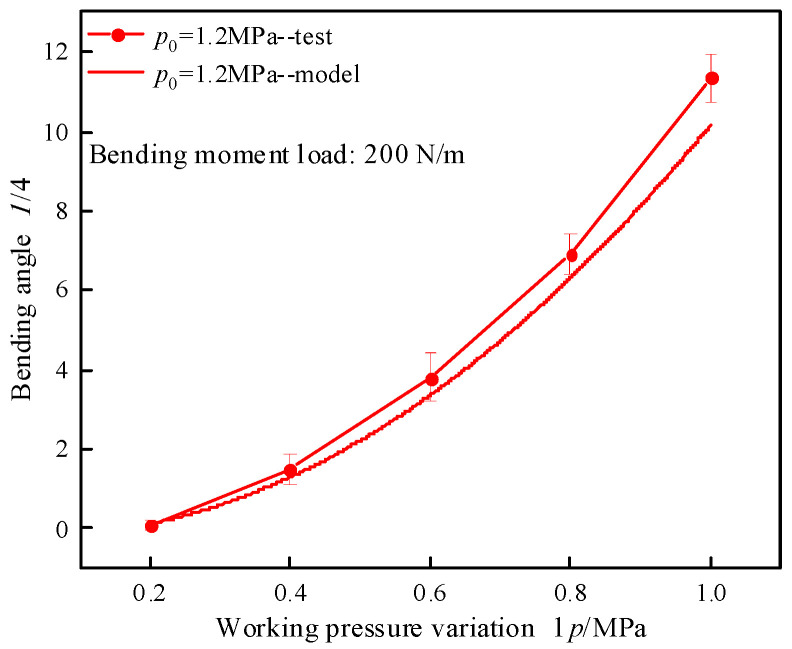
The relationship between bending angle and working pressure variation.

**Figure 6:**
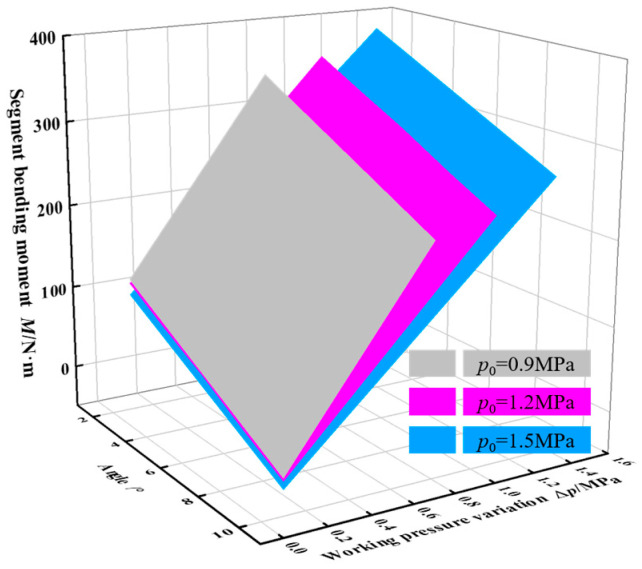
The bending moment of segment.

A flexural test was conducted on a single segmental specimen under an initial operational pressure of 1.2 MPa with an applied bending moment of 200 N·m. The correlation between the pressure variation and bending angle is presented in Figure 5. While discrepancies exist between model and experimental results, the model demonstrates satisfactory fidelity in capturing the bending characteristics of the segment. The maximum bending moments generated by the segmental structure at varying bending angel and initial working pressure were analytically determined using Equation (7), with the computed results presented in Figure 6. The condition for generating the maximum bending moment is derived under the scenario of the maximum working pressure variation across different initial working pressures. Grey, magenta, and blue represent the initial working pressures of 0.9 MPa, 1.2 MPa, and 1.5 MPa, respectively. With the segment bending angle maintained constant, the resultant bending moment exhibited a direct proportionality to incremental changes in operating pressure. Specifically, a pressure increment of 0.1 MPa corresponded to a bending moment variation of 25 N·m. Under constant working pressure variation, the bending moment reduces by approximately 30 N·m for each 2-degree increase in segment bending angle.

### 5.2. Contact Force of Multiple Segments

**Figure 7:**
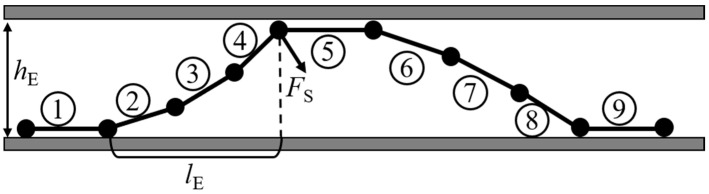
Schematic diagram of S-shaped support for a continuum robot.

Due to the maximum bending angle of the robot segment being less than or equal to 30°, the bending of the segment can be considered equivalent to the rotation of a rod, and the length of the rod can be approximated as the arc length of the segment’s bending, based on the approximation that the sine value of a small angle is approximately equal to the angle value itself. The continuous robot achieves an S-shaped support configuration as illustrated in Figure 7, with the segments simplified as rods. Contact and support are achieved through the continuous deformation of multiple segments and environmental constraints. Taking the diagram as an example, it is assumed that segment 1 is in contact with the lower boundary of the environment and is relatively fixed. Segments 2, 3, and 4 are assumed to be in contact with the upper boundary of the environment through deformation, allowing segment 5 to be in contact with the upper boundary of the environment and be relatively fixed. Segments 6, 7, and 8 are also constrained by deformation and the environmental and lower boundaries, such that segment 9 is in contact with the lower boundary of the environment and is relatively fixed. Segment 1 is equivalent to segment 9. Therefore, a continuous robot composed of multiple segments can perform S-shaped support.

Taking the three-segment S-shaped support (segments 2, 3, and 4) in Figure 7 as an example, segment 4 is subjected to a reaction force constrained by the upper boundary of the environment, with the reaction force acting perpendicular to segment 4. This reaction force will generate a bending moment on segment 2, 3, and 4, which is transmitted to the connection between segment 1 and segment 2, with the most significant bending moment occurring on segment 2. The geometric relationship and moment balance between the S-shaped support and environmental constraints are as follows:

(8) $$h_{E}=l_{2}\cdot \sin\varphi_{2}+l_{3}\cdot \sin(\varphi_{2}+\varphi_{3})+l_{4}\cdot \sin(\varphi_{2}+\varphi_{3}+\varphi_{4})$$

(9) $$l_{E}=l_{2}\cdot \cos\varphi_{2}+l_{3}\cdot \cos(\varphi_{2}+\varphi_{3})+l_{4}\cdot \cos(\varphi_{2}+\varphi_{3}+\varphi_{4})$$

(10) $$M_{2}=F_{S}\cdot \cos(\varphi_{2}+\varphi_{3}+\varphi_{4})\cdot l_{E}$$

where *l* represents the length of the module, *h*_E_ represents the environmental boundary, *l*_E_ represents the force arm that generates a bending moment on the root module due to the contact force, and *M*_2_ represents the bending moment load on segment 2. The geometric relationships and moment balance between the S-shaped support and environmental constraints of continuum robots can be reformulated based on the number of segments.

(11) $$h_{E}=l_{1}\cdot \sin\varphi_{1}+l_{2}\cdot \sin(\varphi_{1}+\varphi_{1})+\cdots +l_{n}\cdot \sin(\varphi_{1}+\varphi_{2}+\cdots +\varphi_{n})$$

(12) $$l_{E}=l_{1}\cdot \cos\varphi_{1}+l_{2}\cdot \cos(\varphi_{1}+\varphi_{2})+\cdots +l_{n}\cdot \cos(\varphi_{1}+\varphi_{2}+\cdots +\varphi_{n})$$

(13) $$M=F_{S}\cdot \cos(\varphi_{1}+\varphi_{2}+\cdots +\varphi_{n})\cdot l_{E}$$

**Figure 8:**
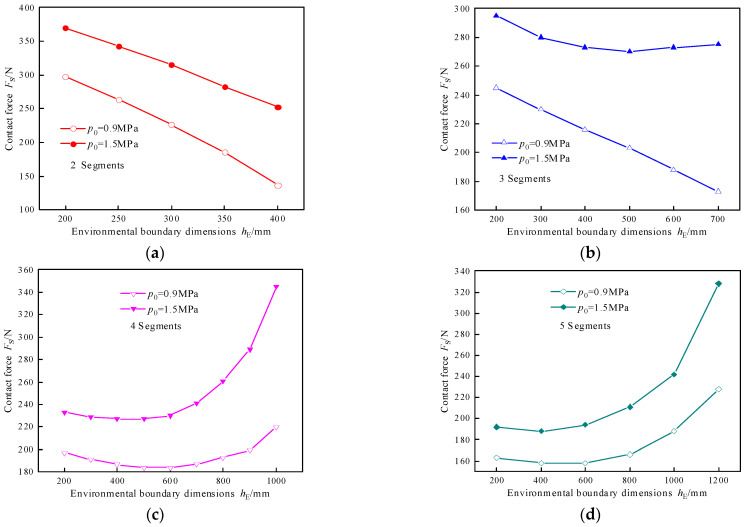
The relationship between contact force and environmental boundary dimensions under different numbers of segments: (**a**) 2-segment S-shaped support, (**b**) 3-segment S-shaped support, (**c**) 4-segment S-shaped support, (**d**) 5-segment S-shaped support.

To ensure the effective support force of the S-shaped support in a continuum robot, the bending angle of the end segment of the continuum robot must not exceed 90°, and the bending angle of a single segment must not exceed 20°. To ensure consistent bending moment load capacity across all segments for achieving maximum contact force, each segment must operate under identical conditions. There are variations in the environmental contact boundaries that S-shaped supports can adapt to depending on the number of segments. Figure 8 illustrates the relationship between contact force and environmental contact size, with the curves representing the results of maximum pressure changes under different initial working pressures.

For two-segment S-shaped support, the adaptive environmental contact boundary increases with the increase in segment bending angle. However, as the force arm of the segments does not change significantly, the contact force decreases with the increase in environmental contact boundary, ranging from 136 N to 370 N. When implementing the three-segment S-shaped support, the trend in contact force varies with the increase in environmental contact boundary under different initial working pressures. At an initial working pressure of 0.9 MPa, the contact force decreases with the increase in environmental contact boundary, similar to the two-segment case. At an initial working pressure of 1.5 MPa, the contact force initially decreases and then exhibits a slight increase as the environmental contact boundary expands. This discrepancy arises because different initial working pressures lead to varying contraction of the segments, resulting in different bending angles of the segments under the same environmental contact boundary. This phenomenon is attributed to the combined effects of segment contraction and the force arm, wherein the contact force lies within the range of 173 N to 295 N. For four- and five-segment S-shaped support, the contact force exhibits a trend of first decreasing and then increasing with the increase in environmental contact boundary under different initial working pressures. The primary cause of the reduction in contact force is that the force arm remains nearly constant within this range, while the bending angle of the segments varies within 5 degrees. As the bending angle increases, the contact force initially decreases. However, with an increase in environmental contact boundary, the segment bending angle also increases. At this point, the force arm decreases sharply with the increase in environmental contact boundary, and the angle between the contact segment and the environmental contact boundary increases, leading to an increase in contact force. The support force achieved by four modules ranges between 183 N and 345 N, while the support force achieved by five modules ranges between 158 N and 328 N.

The primary objective of employing S–shaped support for continuum robots is to achieve relative anchoring with the surrounding environment. This process involves analyzing the magnitude of the contact force to ensure the stable anchoring of the robot. Among the three biomimetic movements, the elephant trunk–inspired biomimetic movement is the most complex and is also the focus of this study.

## 6. Segmental Motion Characteristics of Elephant Trunk Movement

**Figure 9:**
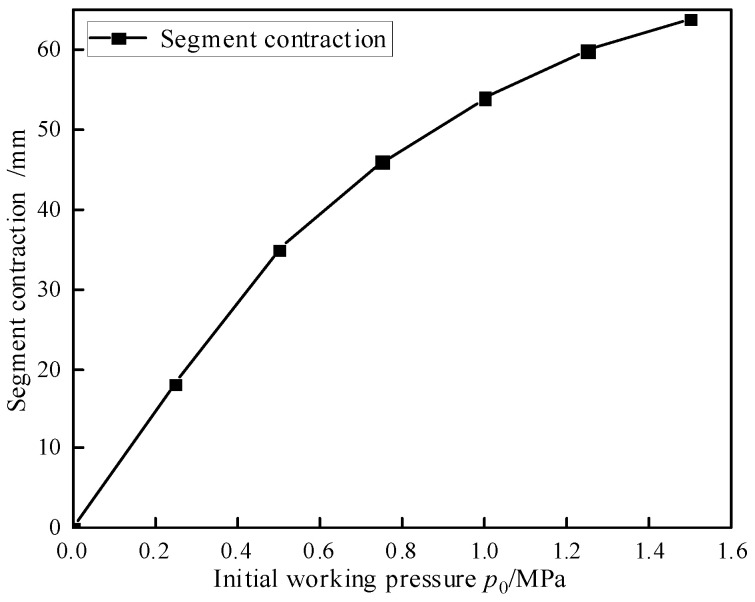
The relationship between segment contraction and initial working pressure.

The segmental movement includes contraction and bending. To determine the range of motion for segmental contraction, an isolated contraction test was conducted. Without any working pressure differential, the initial working pressure was adjusted in increments of 0.25 MPa up to a maximum of 1.5 MPa, and the module contraction was measured. To minimize the risk of accidental errors, the experiments were repeated three times. The relationship between module contraction and initial working pressure *p*_0_ is illustrated in Figure 9, where it can be observed that as the initial working pressure increases, the amount of module contraction decreases. The maximum contraction of the module reaches 64 mm. This phenomenon is attributed to the driving characteristics of WHAMs.

**Figure 10:**
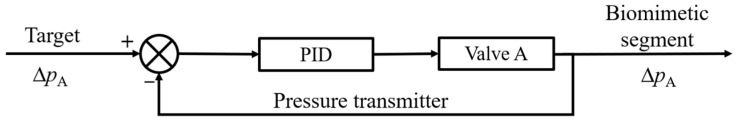
Principle of PID control.

The bending behavior of biomimetic segments can be dissected into two interrelated components: (1) valve-actuated working pressure variations of WHAMs, and (2) working pressure-induced deformations that produce segmental movements. Consequently, the initial priority should be placed on investigating the closed-loop control characteristics governing the working pressure within biomimetic segments. The target was defined as the working pressure differential between the two outlet ports of either pressure difference control valve A or pressure difference control valve B. Theoretically, this pressure differential should equate to twice the magnitude of the WHAM’s working pressure variation from its initial working pressure. The control principles of pressure difference control valve A and pressure difference control valve B are identical. Taking pressure difference control valve A as an example, a PID controller was employed to regulate this pressure differential, and its control architecture is depicted in Figure 10.

During the control process, the following operational sequence was executed:

1. The target differential was compared to the real-time measured operating pressure differential;
2. The discrepancy between these two values was computationally determined;
3. This error signal served as the input for the PID controller;
4. The controller adjusted the valve spool position based on the input signal;
5. Consequent adjustment of the working’s working pressure differential was achieved to converge on the target differential.

The operational position of the total pressure control valve spool was maintained by a spring mechanism. Due to identical valve structures for both pressure difference control valve A and pressure difference control valve B, their PID parameters were uniformly configured. To ensure the effectiveness of PID control, a genetic algorithm was employed to optimize the hyperparameters in the PID controller. Considering the computational complexity and accuracy of genetic algorithms, a target trajectory that ranged from a pressure variation of 0 steps to the maximum pressure variation was set as the evaluation criterion for the genetic algorithm. The specific method for hyperparameter optimization is as follows. The three hyperparameters, *k*_P_, *k*_I_, and *k*_D_, in the PID algorithm were considered as the independent variables of the genetic algorithm, and the squared integral of the difference between the pressure–variation simulation result and the target trajectory was set as the evaluation variable. The optimal individual coefficient was set to 0.4, the population size was set to 20, the maximum evolutionary generation was set to 50, and the fitness function deviation was set to 1 × 10⁻^10^. The optimized PID parameters obtained are shown in Table 4.

Step response tests and sine following experiments were conducted to investigate the working pressure closed-loop control characteristics of biomimetic segments. The average of the two outlet pressures from pressure difference control valve A was designated as the initial operating pressure *p*_0_. Initially, the outlet pressure differential between pressure difference control valve A and pressure difference control valve B was maintained at zero while adjusting the initial operating pressure to the target levels. Subsequently, a PID controller was implemented to stabilize the outlet pressure differential of pressure difference control valve B at zero, followed by closed-loop control characteristic evaluations for pressure difference control valve A’s outlet pressure differential. Three test conditions were established using 0.9 MPa, 0.8 MPa, and 0.7 MPa as initial operating pressures. The experimental scheme is detailed in Table 5.

**Figure 11:**
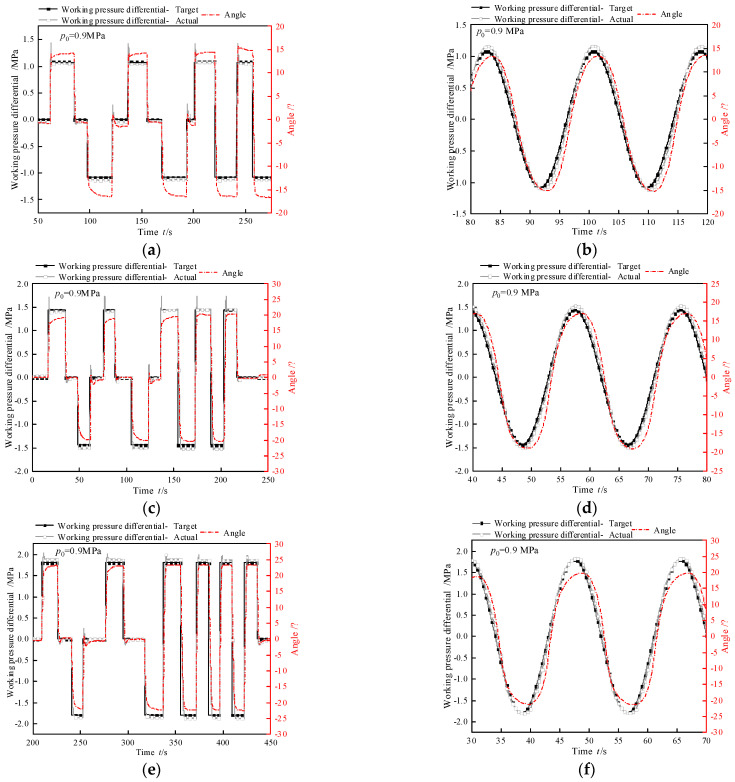

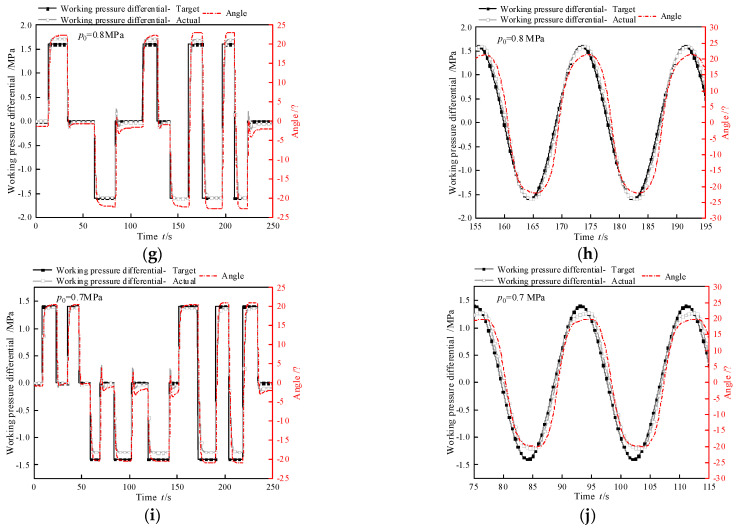
Working pressure closed-loop characteristic test. (**a**) Step test with test condition 1. (**b**) Sine following test with test condition 1. (**c**) Step test with test condition 2. (**d**) Sine following test with test condition 2. (**e**) Step test with test condition 3. (**f**) Sine following test with test condition 3. (**g**) Step test with test condition 4. (**h**) Sine following test with test condition 4. (**i**) Step test with test condition 5. (**j**) Sine following test with test condition 5.

Experimental results for the step response test and sine following test are presented in Figure 11. The corresponding closed-loop dynamic characteristics of the working pressure are quantified in Table 6 and Table 7. In the step response test, under identical initial operating pressures, the adjustment time diminishes with decreasing step amplitudes, while the overshoot magnitude correspondingly increases. This phenomenon arises due to the stationary valve spool of the intermediate valve maintaining a consistent flow capacity. During step amplitude reduction, the hydraulic fluid volume required for complete deformation of the hydraulic artificial muscle decreases. Consequently, under constant flow capacity, smaller step amplitudes correlate with shorter adjustment times but greater overshoot.

To illustrate, consider pressure difference control valve A under three conditions as follows: Initial working pressure = 0.9 MPa, step amplitude = 1.8 MPa; initial working pressure = 0.8 MPa, step amplitude = 1.6 MPa; initial working pressure = 0.7 MPa, step amplitude = 1.4 MPa (denoted as maximum step amplitude). With decreasing initial pressures at maximum step amplitudes, adjustment time increases. This behavior stems from two interrelated factors: valve spool position dependency, where lower initial pressures correspond to smaller spool openings, reducing the valve’s effective flow area. Conversely, higher initial pressures indicate larger spool openings and enhanced flow capacity. Pressure differential dynamics, where the rate of pressure change in hydraulic artificial muscles depends on the pre-to-post pressure differential. Larger differentials expedite fluid flow, shortening the pressure transition time. Collectively, these mechanisms prolong the adjustment time under maximum step amplitudes as initial pressure decreases.

In the sine following test, as the initial working pressure increases, the phase lag of the tracking error also increases with the increase in amplitude. This is because the valve spool of the total pressure control valve remains stationary and its flow capacity remains unchanged. However, as the sine wave amplitude increases, the rate of change of the set pressure difference at the outlet of pressure difference control valve A per unit time increases, while the flow characteristics of the valve remain unchanged. Therefore, as the amplitude increases, the tracking error and phase lag increase synchronously. At the maximum amplitude, as the initial working pressure decreases, the tracking error decreases while the phase lag increases. As the total pressure control valve core moves, its outlet flow characteristics change, resulting in an increase in phase lag. However, at the same time, as the sine amplitude decreases, the rate of change in the set value of the internal valve outlet pressure difference between units also decreases. The combined effect of the two leads to a decreasing trend in tracking error. The sine following test with different periods are shown in Table 8, which proves that the segments can be master–slave controlled at low frequencies.

## 7. Master–Slave Control of Elephant Trunk Movement

### 7.1. Master Controller Design

**Figure 12:**
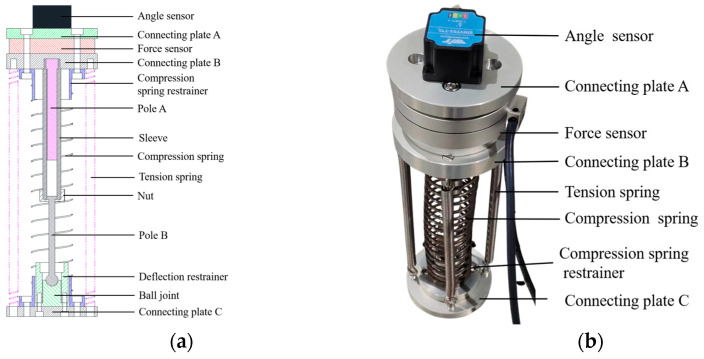
Master controller. (**a**) Structure of master controller and (**b**) prototype of master controller.

Based on the design of an isomorphic master–slave control system, the structure of the master controller is depicted in Figure 12a. Four tension springs are used to simulate WHAMs, while the compression spring in the master control unit mimics the spring of the biomimetic segment. Connecting plate C is used to install a ball joint and deflection restrainer, and the ball joint is connected to one end of pole B. The other end of pole B extends into the sleeve and is retained by a nut to prevent it from slipping out. The sleeve is connected to connecting Plate B and incorporates pole A, which is fixed in position relative to both the connecting plate B and the sleeve. One side of connecting plate B is connected to the compression spring restrainer, and the other side is connected to the force sensor. The other side of the force sensor is connected to the connecting plate A, and the angle sensor is installed on the connecting plate A to measure the deflection angle of the master control unit. Based on the motion range of the module, the axial motion range of the isomorphic master control unit is selected to be slightly greater than half of the module’s maximum contraction, and the deflection angle is marginally larger than the module’s maximum deflection angle. The axial contraction of the master controller is defined as the distance measured between the bottom of pole A and the top of pole B. The bending angle of the master controller is determined by the interaction between pole B and the deflection restrainer. The prototype of the master controller is depicted in Figure 12b. The angle sensor is employed to measure the deviation angle in two directions, while the force sensor is utilized to assess the axial contraction of the master control unit. The technical parameters of the angle sensor and force sensor are shown in Table 9.

### 7.2. Master–Slave Workspace Mapping

**Figure 13:**
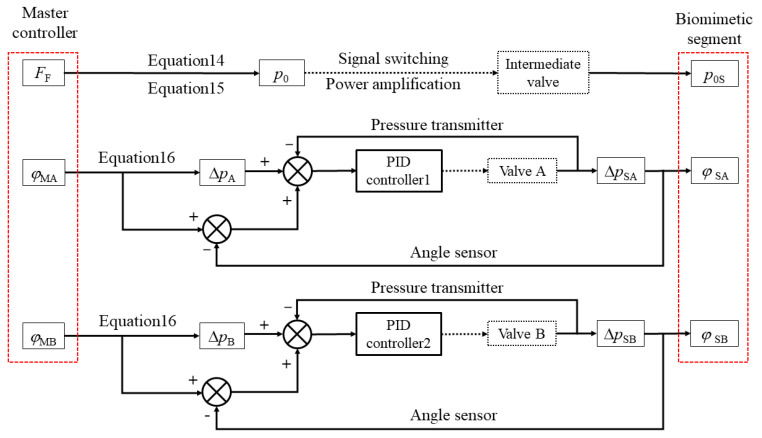
The principle of PID control in the master–slave system.

In the natural state, due to the balancing effect of the compression spring and the tension spring, the master controller unit does not bend, and the force detected by the force sensor is close to 0 N. Assuming that the axial load of the compression spring during the operation of the master controller remains consistent with its pure compression state and is solely dependent on the compression. Therefore, the contraction of the master controller can be expressed as:

(14) $$\Delta L_{M}=F_{F}/(k_{C}+4k_{T})$$

where, Δ*L*_M_ is the contraction of the master control unit, *k*_C_ is elastic coefficient of the compression spring, *k*_T_ is elastic coefficient of the tension spring, and *F*_F_ is the axial load of the force sensor. By combining Jia et al.’s work, it can be concluded that there is a relationship between the initial working pressure of WHAMs and the load measured by the master control unit’s force sensor:

(15) $$p_{0}=\frac{k_{\mathrm{spring}}\cdot (h_{0}+2\Delta L_{M})}{\pi D_{0}^{2}[a{\left(1-2\Delta L_{M}/L_{0}\right)}^{2}-b]}$$

(16) $$\Delta p=\frac{\varphi [1+k_{3}p_{0}r^{2}(1-\varepsilon_{C})]+k_{2}\varepsilon_{C}}{k_{1}\{a[{\left(1-\varepsilon_{C}\right)}^{2}]-b\}\cdot r}$$

where *k*_1_, *k*_2_, and *k*_3_ are correction coefficients. In summary, the mapping relationship between the master–slave system and control algorithm are shown in the Figure 13. The control algorithm utilized in this study is implemented by a proportional–integral–derivative (PID) controller. The parameters *F*_F_, *β*_M1_, and *β*_M2_ in the master controller unit are measured by force sensor and angle sensor, while the parameters *p*_0S_, Δ*p*_SA_, Δ*p*_SB_, *φ*_SA_, and *φ*_SB_ in the biomimetic segment are measured by pressure sensors and angle sensors.

A genetic algorithm was also employed for hyperparameter optimization of a cascade PID control system. It is important to note that, to ensure satisfactory optimization results, the optimization process was divided into two stages. In the first stage, the genetic algorithm was utilized to optimize the PID parameters of the inner loop for working pressure. The evaluation condition was based on the target motion from a step change in working pressure to the maximum pressure change, with the square integral of the difference between the simulated pressure change and the target set as the evaluation metric. In the second stage, the optimization of the PID parameters for the angle outer loop was performed. The optimized PID parameters of the working pressure inner loop were incorporated into the simulation model as known variables, and the PID parameters of the angle outer loop were treated as independent variables in the genetic algorithm. The optimized PID parameter results are presented in Table 10.

The force detected in the master controller can be used to calculate the initial working pressure p₀ required for the biomimetic segment to achieve the corresponding contraction through Equations (14) and (15). The total pressure control pressure difference control valve adjusts the initial working pressure setting of the biomimetic segment via open-loop control. In addition, the two detected tilt angles in the master controller can be used as target values to calculate the pressure change required to reach this tilt angle, i.e., the pressure difference, according to Equation (16). Then, using PID controller 1, the working pressure difference of a set of hydraulic artificial muscles quickly approaches the calculated result, and PID controller 2 is utilized to reduce the pose deviation between the master controller and the biomimetic segment.

**Figure 14:**
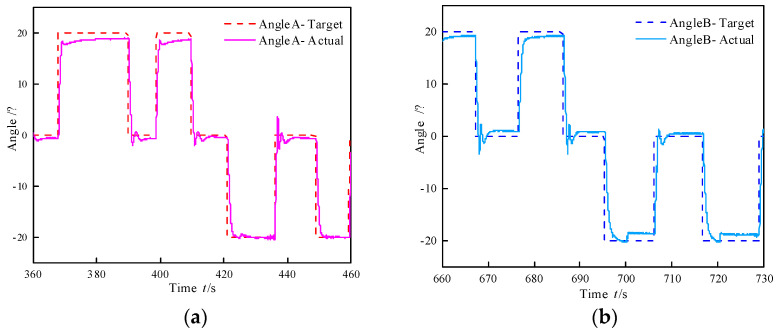
Step test on bending angle of biomimetic segment. (**a**) Pressure difference control valve A. (**b**) Pressure difference control valve B.

To investigate the motion characteristics of the biomimetic segment, step and sine following tests were conducted on its bending angle, with an amplitude of 20°. The results of the bending angle step experiment for the biomimetic segment are illustrated in Figure 14, revealing that the step responses in the two directions are inconsistent. This phenomenon is attributed to the connection between the differential pressure control valve and the runner plate. As illustrated in Figure 1c, one end of the spool of the pressure difference control valve is positioned within the return water chamber. Consequently, during the movement of the spool, it is continuously subjected to a force exerted by the return water pressure. Specifically, for angle A, the steady-state error is 1.5° and the settling time is 9.31 s under a positive step input, whereas under a negative step input, the steady-state error reduces to 0.08° and the settling time shortens to 4.31 s. For angle B, the steady-state error is 0.73° and the settling time is 6.01 s under a positive step input, and it increases to 1.19° with a settling time of 4.44 s under a negative step input.

**Figure 15:**
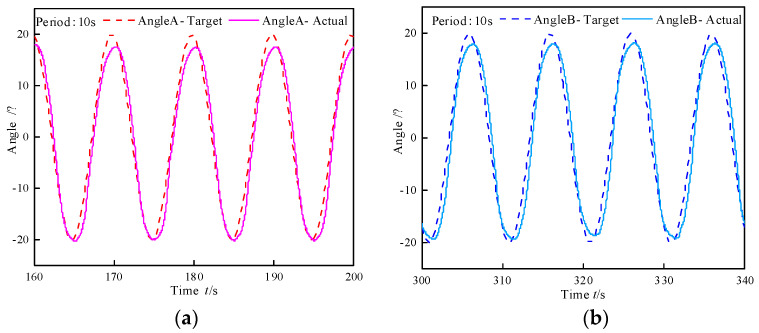
Sine following test on bending angle of biomimetic segment. (**a**) Pressure difference control valve A. (**b**) Pressure difference control valve B.

Sine wave analyses following experiments with various periods were conducted to analyze the bending motion characteristics of biomimetic segments. Taking a 10 s period as an example, the sine wave following characteristics of angles A and B are depicted in Figure 15. Similarly, discrepancies exist in the sine wave following characteristics between the positive and negative directions. The tracking error and phase lag of angles A and B at a set value of 0° under different cycles are presented in Table 11. It can be noted that with an increase in the period, the tracking error and phase lag associated with angles A and B exhibit a decreasing trend. This phenomenon arises from the fact that the valve core of the total pressure control valve maintains a constant flow capacity when its operating position remains stable. As the duration of the sine cycle lengthens, the volumetric change necessary for the deformation of WHAMs per unit time diminishes. In other words, the required flow rate declines, which subsequently leads to a reduction in both the tracking error and phase lag.

**Figure 16:**
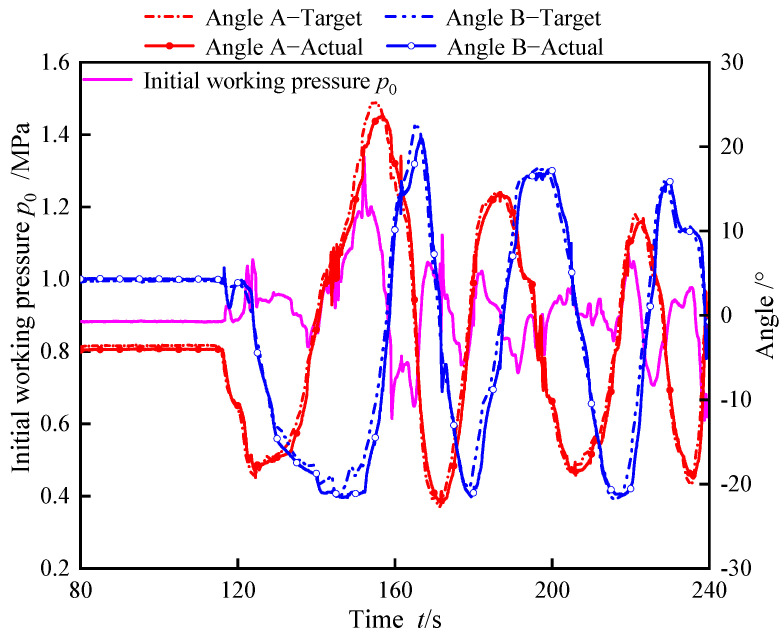
Master-slave control test.

The master–slave control test process is illustrated in Figure 16. The target angle is measured by the angle sensor of the master controller, while the actual angle is measured by the angle sensor integrated into the biomimetic segment. During the experiment, the operator manipulated the master controller, and the angles A and B measured by the angle sensor within the master controller were employed as target signals for input into the control program. The biomimetic segment mimicked the motion of the master controller, as depicted in Figure 16. The maximum values of the two deflection angles controlled by pressure difference control valve A were 23.5° and 22.1°, respectively, while the maximum values of the two deflection angles controlled by pressure difference control valve B were 21.0° and 21.3°, respectively. In addition, during the experiment, the initial working pressure varied within the range of 0.615–1.34 MPa, and both the maximum and minimum values of the initial working pressure occurred when the bending angle of the two degrees of freedom was at a large angle. At this point, the valve cores of pressure difference control valve A and pressure difference control valve B deviated from the center position and moved to both ends. The hydraulic resistance of pressure difference control valve A and pressure difference control valve B synchronously changed, thereby altering the local hydraulic network formed by their parallel connection, resulting in a significant change in the initial working pressure. In the master–slave control experiment, it is also necessary to evaluate the control accuracy, stability, and other performance metrics of the master–slave control system. When the master controller’s angle A reaches the maximum angle, the bending angle deviation between the master controller and the biomimetic segment peaks at a maximum error of 3.01°. Similarly, when the master controller’s angle B reached the maximum angle, the angle deviation between the master controller and the integrated valve control module reached a maximum error of 3.93°. The primary sources of error in the bending angle between the master controller and the biomimetic segment were the mechanical delay and communication latency during motion execution. Taking angle A controlled by pressure difference control valve A as an example, where the deflection angle of the master controller is captured by the tilt angle sensor and fed into the system, the required pressure change is calculated using Equation (16). PID controller 1 then actuates the spool of pressure difference control valve A to adjust the WHAMs’ working pressures, achieving segment motion. Concurrently, PID controller 2 synchronizes the biomimetic segment’s motion to approximate the master controller’s input angle. Thus, errors in the master–slave system primarily stem from mechanical hysteresis. Additionally, signals from the master controller must undergo computation and transmission into the control system, inevitably introducing communication delays. These two factors collectively contribute to the observed errors. During experiments, local oscillations in the biomimetic segment’s bending angle caused fluctuations in the initial working pressure. However, the master–slave system maintained stable operation, ensuring overall system stability.

## 8. Conclusions

In this study, a three-degree-of-freedom biomimetic segment driven by WHAMs and supported by springs was proposed, achieving integrated and modular design. The continuum robot composed of this segment can execute biomimetic motion modes based on operational environmental constraints. By studying the motion characteristics of a single segment and using an isomorphic master–slave control system for control, the following conclusions are drawn:

1. The continuum robot composed of this segment can execute earthworm-, snake-, and elephant trunk-biomimetic motion modes based on operational environmental constraints. During long-distance operational tasks, distinct segments of the continuum robot can adopt varying biomimetic configurations to meet specific requirements;
2. A single biomimetic segment can achieve a maximum contraction of 64 mm and a maximum bending angle of 23 degrees. When the initial working pressure is 1.2 MPa and the change in working pressure is 1.0 MPa, the biomimetic segment can still output a bending moment of 200 N·m or more at a bending angle of 10 degrees;
3. An isomorphic master controller was designed based on the motion range of a single segment, and a mapping relationship between the master controller and the biomimetic segment was established. Open-loop control was applied to the total pressure control valve, and PID closed-loop control was performed on pressure difference control valve A and pressure difference control valve B to achieve pose control of the isomorphic master–slave control system. The maximum bending angle deviation between the master controller and the biomimetic segment does not exceed 4°, and the system exhibits good stability;
4. In the biomimetic segment proposed in this article, the integration of the control valve within the segment results in a significant coupling effect among its three degrees of freedom. The total pressure control valve limits the flow to both pressure difference control valve A and pressure difference control valve B. To enhance the real-time responsiveness of biomimetic segments, it is crucial to enhance the valve control system to better support real-time operations.

## Figures and Tables

**Table 1:** The physical parameters of the segment.

**Segment**	Maximum diameter	185 mm
	Maximum length	480 mm
**WHAM**	Center distance	75 mm
	Initial diameter	30 mm
	Initial working length	300 mm
	Initial braided angle	28°
**Spring**	Pitch diameter	75 mm
	Wire diameter	14 mm
	Uncompressed height	450 mm
	Pre-compression	10 mm
	Active number of coils	15

**Table 2:** The relevant coefficients of the model.

Coefficients	Value
*k*_s_	3.153
*k*_τ_	0.024
*k*_F_	0.963
*k*_ε_	1.184
*k*_spring_	62.930 N/mm
*G*	8.293 × 10^4^ MPa
*a*	13.799
*b*	5.616

**Table 3:** The technical parameters of the force sensor.

Sensor	Maximum Range	Linearity	Signal
Force sensor	2000 N	0.02% FS	0–5 V
Angle sensor	30°	0.01% FS	0–5 V
Pressure transmitter	6 MPa	0.01% FS	0–10 V

**Table 4:** PID controller parameters.

*k*_P_	*k*_I_	*k*_D_
15	0.1	5

**Table 5:** Experimental scheme of closed-loop pressure control characteristics.

Case	Initial Working Pressure	Working Pressure Differential
Test condition 1	0.9 MPa	1.08 MPa
Test condition 2		1.44 MPa
Test condition 3		1.8 MPa
Test condition 4	0.8 MPa	1.6 MPa
Test condition 5	0.7 MPa	1.4 MPa

**Table 6:** Experimental results of the step response experiment.

Initial Working Pressure	Step Amplitude	Steady-State Value	Steady-State Error	Overshoot	Settling Time
0.7 MPa	+1.4 MPa	+1.38 MPa	0.02 MPa		6.92 s
	−1.4 MPa	−1.27 MPa	0.13 MPa		4.65 s
0.8 MPa	+1.6 MPa	+1.71 MPa	0.11 MPa		3.36 s
	−1.6 MPa	−1.58 MPa	0.02 MPa		4.25 s
0.9 MPa	+1.8 MPa	+1.88 MPa	0.08 MPa	7.18%	2.81 s
	−1.8 MPa	−1.85 MPa	0.05 MPa		4.22 s
	+1.44 MPa	+1.42 MPa	0.02 MPa	21.83%	1.83 s
	−1.44 MPa	−1.50 MPa	0.06 MPa		1.31 s
	+1.08 MPa	+1.05 MPa	0.03 MPa	37.14%	1.15 s
	−1.08 MPa	−1.13 MPa	0.05 MPa		0.87 s

**Table 7:** Experimental results of the sine following test.

Initial Working Pressure	Period	Amplitude	Tracking Error	Phase Lag
*p*_0_ = 0.7 MPa	17 s	1.4 MPa	0.172 MPa	7.6°
*p*_0_ = 0.8 MPa	17 s	1.6 MPa	0.18 MPa	7.2°
*p*_0_ = 0.9 MPa	17 s	1.08 MPa	0.11 MPa	5.9°
		1.44 MPa	0.13 MPa	6.1°
		1.8 MPa	0.21 MPa	6.4°

**Table 8:** Experimental results of the sine following test with different periods.

Initial Working Pressure	Amplitude	Period	Tracking Error	Phase Lag
*p*_0_ = 0.9 MPa	1.8 MPa	5 s	0.24 MPa	7.5°
		10 s	0.23 MPa	7.1°
		15 s	0.22 MPa	6.7°
		20 s	0.19 MPa	4.5°
		25 s	0.16 MPa	4.5°
		30 s	0.13 MPa	3.1°

**Table 9:** The technical parameters of sensor in the master controller.

Sensor	Maximum Range	Linearity	Signal
Angle sensor	30°	0.01% FS	0–5 V
Force sensor	50 N	0.01% FS	0–10 V

**Table 10:** The PID controller parameters of the master–slave control system.

PID Controller 1			PID Controller 2		
*k*_P_	*k*_I_	*k*_D_	*k*_P_	*k*_I_	*k*_D_
10	0.1	1	0.07	0	0.01

**Table 11:** Sine following test on the bending angle of the biomimetic segment with different periods.

Amplitude	Period	Angle A		Angle B	
		Tracking Error	Phase Lag	Tracking Error	Phase Lag
20°	5 s	3.5°	9.4°	5.8°	14.4°
	10 s	3.1°	8.3°	4.3°	10.8°
	15 s	2.6°	7.3°	3.2°	8.6°
	20 s	2.4°	6.1°	2.6°	6.3°
	25 s	2.2°	4.5°	2.3°	5.8°
	30 s	1.9°	3.1°	1.9°	4.9°

## Data Availability

Data are contained within the article.

## References

[1] Ren K., Yu J. (2021). Research status of bionic amphibious robots: A review. Ocean Eng..

[2] Afzal N., Rehman M., Seneviratne L., Hussain I. (2024). The Convergence of AI and animal-inspired robots for ecological conservation. Ecol. Inform..

[3] Sachsenmeier P. (2016). Industry 5.0—The relevance and implications of bionics and synthetic biology. Engineering.

[4] Lu G., An N., Liu Z. Design and kinematics analysis of a bionic finger hand rehabilitation robot mechanism. Proceedings of the 2019 34rd Youth Academic Annual Conference of Chinese Association of Automation (YAC).

[5] Ni P., Sun J., Dong J. (2024). Design and Control of an Upper Limb Bionic Exoskeleton Rehabilitation Device Based on Tensegrity Structure. Appl. Bionics Biomech..

[6] Dong D., Wang Z., Guan J., Xiao Y., Wang Y. (2025). Research on key technology and application progress of rescue robot in nuclear accident emergency situation. Nucl. Eng. Technol..

[7] Li J., Zhou X., Gui C., Yang M., Xu F., Wang X. (2025). Adaptive climbing and automatic inspection robot for variable curvature walls of industrial storage tank facilities. Autom. Constr..

[8] Vo A.T., Truong T.N., Kang H.J., Nguyen N.H.A. (2025). Prescribed Performance Model-Free Sliding Mode Control Using Time-Delay Estimation and Adaptive Technique Applied to Industrial Robot Arms. Inf. Sci..

[9] Liu X., Zhang M., Liu W. Methods to Modular Robot Design. Proceedings of the 2008 Second International Symposium on Intelligent Information Technology Application.

[10] Tang S., Yao J., Yu Y., Zhao G. (2025). An Extendable and Deflectable Modular Robot Inspired by Worm for Narrow Space Exploration. Actuators.

[11] Mu Z., Wang H., Xu W., Liu T., Wang H. (2017). Two types of snake-like robots for complex environment exploration: Design, development, and experiment. Adv. Mech. Eng..

[12] Fang H., Wang C., Li S., Xu J., Wang K.W. Design and experimental gait analysis of a multi-segment in-pipe robot inspired by earthworm’s peristaltic locomotion. Proceedings of the Bioinspiration, Biomimetics, and Bioreplication 2014.

[13] Wang N., Zhang Y., Zhang G., Zhao W., Peng L. (2022). Development and Analysis of Key Components of a Multi Motion Mode Soft-Bodied Pipe Robot. Actuators.

[14] Jiang J., Zhang F., Wang L. (2024). Soft modular pipe robot inspired by earthworm for adaptive pipeline internal structure. Smart Mater. Struct..

[15] Wang X., Bilsky M., Bhattacharya S. (2021). Search-based configuration planning and motion control algorithms for a snake-like robot performing load-intensive operations. Auton. Robot..

[16] Virgala I., Kelemen M., Prada E., Sukop M., Kot T. (2021). A snake robot for locomotion in a pipe using trapezium-like travelling wave. Mech. Mach. Theory.

[17] Huang Q., Wang P., Wang Y., Xia X., Li S. (2022). Kinematic analysis of bionic elephant trunk robot based on flexible series-parallel structure. Biomimetics.

[18] Yang F., Huang H., Chen Y., Li W., Lai Q., Ma R. Research and verification of robot master-slave control algorithm for nuclear power maintenance scenarios. Proceedings of the 15th International Conference on Intelligent Robotics and Applications (ICIRA).

[19] Ai Y., Pan B., Niu G., Fu Y., Wang S. Master-slave control technology of isomeric surgical robot for minimally invasive surgery. Proceedings of the 2016 IEEE International Conference on Robotics and Biomimetics (ROBIO).

[20] Wang L., Zhang L., Wang G., Chen X., Yang S., Jia P. Study on master-slave deepwater manipulator. Proceedings of the OCEANS 2017—Aberdeen.

[21] Zheng Z., Guan Y., Su M., Wu P., Hu J., Zhou X., Zhang H. Development of isomorphic master-slave robots with modular method. Proceedings of the 2013 IEEE International Conference on Information and Automation (ICIA).

[22] Kishi T., Ikeuchi M., Nakamura T. (2015). Development of a peristaltic crawling inspection robot for half-inch pipes using pneumatic artificial muscles. SICE J. Control Meas. Syst. Integr..

[23] Zhao Y.W., Huang H.R., Yuan W.Z., Liu X.M., Cao C.C. (2024). Worm-Inspired, Untethered, Soft Crawling Robots for Pipe Inspections. Soft Robot..

[24] Kandhari A., Wang Y.F., Chiel H.J., Quinn R.D., Daltorio K.A. (2021). An Analysis of Peristaltic Locomotion for Maximizing Velocity or Minimizing Cost of Transport of Earthworm-Like Robots. Soft Robot..

[25] Li Q., Zhao W.Y. (2023). Design of a Modular Pipeline Robot Structure and Passing Ability Analysis. IEEE Access.

[26] Jia Y., Zhang Z., Yang Y., Xu W., Yang R., Gong Y. (2024). Static characteristics of a biomimetic robot module driven by water hydraulic artificial muscles resisted with helical spring. Sens. Actuators A Phys..

